# Metabo-tip: a metabolomics platform for lifestyle monitoring supporting the development of novel strategies in predictive, preventive and personalised medicine

**DOI:** 10.1007/s13167-021-00241-6

**Published:** 2021-05-04

**Authors:** Julia Brunmair, Andrea Bileck, Thomas Stimpfl, Florian Raible, Giorgia Del Favero, Samuel M. Meier-Menches, Christopher Gerner

**Affiliations:** 1 Department of Analytical Chemistry, Faculty of Chemistry, University Vienna, Vienna, Austria; 2 Joint Metabolome Facility, University and Medical University of Vienna, Vienna, Austria; 3 Department of Laboratory Medicine, Medical University of Vienna, Vienna, Austria; 4 Max F. Perutz Laboratories, University of Vienna, Vienna, Austria; 5 Research Platform “Rhythms of Life”, University of Vienna, Vienna, Austria; 6 Department of Food Chemistry and Toxicology, Faculty of Chemistry, University of Vienna, Vienna, Austria

**Keywords:** Exposomics, Histamine, Individualised metabolomics, Lifestyle, Mass spectrometry, Metabo-tip, Molecular patterns, Multi-omics, Predictive preventive personalised medicine (PPPM), Risk assessment, Sweat, Xenobiotics

## Abstract

**Background/aims:**

Exposure to bioactive compounds from nutrition, pharmaceuticals, environmental contaminants or other lifestyle habits may affect the human organism. To gain insight into the effects of these influences, as well as the fundamental biochemical mechanisms behind them, individual molecular profiling seems to be a promising tool and may support the further development of predictive, preventive and personalised medicine.

**Methods:**

We developed an assay, called metabo-tip for the analysis of sweat, collected from fingertips, using mass spectrometry—by far the most comprehensive and sensitive method for such analyses. To evaluate this assay, we exposed volunteers to various xenobiotics using standardised protocols and investigated their metabolic response.

**Results:**

As early as 15 min after the consumption of a cup of coffee, 50 g of dark chocolate or a serving of citrus fruits, significant changes in the sweat composition of the fingertips were observed, providing relevant information in regard to the ingested substances. This included not only health-promoting bioactive compounds but also potential hazardous substances. Furthermore, the identification of metabolites from orally ingested medications such as metamizole indicated the applicability of this assay to observe specific enzymatic processes in a personalised fashion. Remarkably, we found that the sweat composition fluctuated in a diurnal rhythm, supporting the hypothesis that the composition of sweat can be influenced by endogenous metabolic activities. This was further corroborated by the finding that histamine was significantly increased in the metabo-tip assay in individuals with allergic reactions.

**Conclusion:**

Metabo-tip analysis may have a large number of practical applications due to its analytical power, non-invasive character and the potential of frequent sampling, especially regarding the individualised monitoring of specific lifestyle and influencing factors. The extraordinarily rich individualised metabolomics data provided by metabo-tip offer direct access to individual metabolic activities and will thus support predictive preventive personalised medicine.

**Supplementary Information:**

The online version contains supplementary material available at 10.1007/s13167-021-00241-6.

## Introduction

It is now widely known that lifestyle choices are pivotal for a person’s health and life quality, and it is estimated that at least 40% and up to 95% of chronic illnesses can be traced back to lifestyle risk factors (e.g. smoking, lack of physical activity and dietary habits) [1, 2]. Moreover, a positive correlation between the adoption of healthy lifestyle choices and a reduced risk of mortality as well as postponing or even avoiding many types of chronic illnesses such as cancer, cardiovascular diseases and metabolic syndrome has been demonstrated [1, 3–8]. Insulin resistance, abnormal lipid metabolism, diabetes and hypertension are risk factors highly correlated with obesity [9–11], while smoking is known to cause several cancers, especially of the lung and upper airways [12, 13]. In contrast, regular physical activities as well as healthy eating habits such as the Mediterranean diet [14] have already shown to reduce the risk of several chronic conditions such as cardiovascular disease [15, 16], to play a protective role in cancer prevention [17], to promote longevity [18] and to decrease the risk of developing metabolic syndrome and type 2 diabetes [19]. Observational studies suggest that lifestyle changes, mainly dietary [20], can improve the immune system and reduce the risk of recurrence of certain types of cancers, such as ovarian cancer [21]. Therefore, fast and efficient monitoring of biomarkers reflecting individual lifestyle patterns could play a preventive role in the development of chronic disorders.

### Monitoring of individual metabolic responses to xenobiotic exposures

The investigation of metabolic response to environmental exposures is an emerging field of research in toxicology [22, 23]. The so-called exposome includes exogenous exposure not only to the environment, diet or lifestyle factors but also to biological processes reflecting internal responses to exposure [24–26]. For example, a chronic low-dose exposure to mycotoxins, which are frequently detected as natural contaminants in foods, has already been associated with the onset of various diseases. The analysis of mycotoxins as biomarkers for exposure to contaminated food was successfully performed using plasma, serum, urine and milk samples [27, 28]. The origin of many metabolites, which are either endogenously produced, originate from the gut microbiome, or come from the environment via nutrition or smoking, is already being investigated in great detail [29]. In particular, serum metabolomics has successfully revealed several biomarkers which have improved our understanding of disease mechanisms and which are being used in clinical settings for the diagnosis of diseases as well as in the monitoring of therapeutic outcomes [30].

### A fast and non-invasive sweat collection procedure in humans hyphenated with an ultra-sensitive LC–MS/MS approach

A comprehensive analysis of endogenous processes related to the uptake and individual metabolism of xenobiotics requires sensitive analytical techniques in addition to non-invasive and fast sampling methods, allowing short interval sampling and therefore enabling kinetic time-course measurements in humans [31, 32]. The analysis of sweat from the fingertips fulfils these requirements and supports compliance of test subjects. Over decades, fingerprints have been used to identify individuals and more recently play an important role in lifestyle monitoring via imaging mass spectrometry [33]. Exogenous compounds found in bug sprays and sunscreens as well as food oils, alcohols and citrus fruits were detected in fingerprints, offering relevant chemical information about the tested person [34]. Moreover, fingerprints are used to detect illicit drugs and their metabolites [35, 36]. In contrast to the investigation of fingerprints, the analysis of sweat has been successfully proven in diagnostic medicine to enable the monitoring of individual metabolic and health states [37, 38]. Sweat is mainly composed of water (99%), but includes also numerous substances such as electrolytes, lactate, pyruvate, urea, amino acids, proteins, peptides, fatty acids, hormones and xenobiotics (e.g. cosmetics, medications and drugs including ethanol) [39]. Antibodies and cytokines detected in sweat may serve as potential biomarkers for diseases [38] and, as already demonstrated, for disease states in cystic fibrosis [40] and active tuberculosis [41]. Moreover, cortisol has been successfully quantified in human eccrine sweat, demonstrating the potential of finger sweat analysis in regard to monitor endogenous processes related to stress [42].

### The power of individual metabolic profiling in the context of PPPM

Only recently, we successfully demonstrated kinetic time-course measurements of metabolic activities in humans, indicating that finger sweat analyses may become a valuable tool for precision medicine [32]. In contrast to other minimally invasive approaches [43, 44], the presented non-invasive metabolomics assay works quickly and easily while offering tremendous investigative power. We have termed it “metabo-tip”. Indeed, the ingestion of many bioactive compounds contained in food may become detectable via metabo-tip within minutes after consumption. Applying mathematical modelling strategies, it was possible to overcome critical normalisation challenges and to obtain quantitative measures for individual metabolic properties [32]. In this project, we investigated metabo-tip in regard to monitoring lifestyle parameters such as the presence of endogenous and exogenous bioactive compounds or exposure to toxins contained in foods or beverages. These parameters could be described by time-dependent metabolic patterns detected in sweat from the fingertip. This basic research study was conducted to explore potential fields of application of metabo-tip analysis in the clinical routine, thereby supporting the further development of predictive, preventive and personalised medicine (PPPM). Thus, the potential of metabo-tip analysis in order to monitor individual metabolic responses upon the uptake of xenobiotics and potentially unexpected toxins was investigated. The observation of metabolic diurnal rhythms and distinct individual responses to potentially adverse exposure promises successful future applications of metabo-tip analysis not only in the general assessment of individual lifestyle parameters but also in the context of PPPM.

## Material and methods

### Reagents and chemicals

LC–MS grade formic acid, methanol and water used for chromatographic separation as well as for preparation of internal standards and samples were purchased from VWR (Germany). Xenobiotic and metabolic standards were obtained either from Sanova Pharma GmbH (Austria), from Sigma-Aldrich (Austria), or from Honeywell Fluka (GER). Filter papers (standardised to 1 cm^2^) used for sampling were stamped out of fuzz-free paper from Kimtech Science (USA).

### Cohort design

Several volunteers were recruited after giving written, informed consent for the different studies as outlined in Table 1. These experiments were approved by the ethical committee of the University of Vienna (no. 00337). Volunteers may be part of more than one study (A–E). Gender distribution of participants was equally balanced between male and female and their ages ranged from 20 to 50 years. Some studies (C–E) required a fasting period of 12 h for caffeinated foods (chocolate, energy bars) and beverages (coffee, tea, energy drinks) before beginning with the experiment in order to avoid interferences with the investigation of xenobiotic metabolism. Finger sweat samples were collected just before donors consumed a 50-g chocolate bar or a double espresso (0 min). Finger sweat samples were thereafter collected in short intervals as outlined in Table 1. In studies A and B, the general lifestyle of volunteers was monitored. There were no dietary restrictions placed on the test subjects.

**Table 1:** Overview of all studies discussed in this publication

Study	Participants	Design	Sampling time points	Restrictions
A	6 participants	30 consecutive days of sampling	2 × per day (morning and evening)	No restrictions
B	10 participants	10 consecutive days of sampling	3 × per day (morning, lunch, evening)	No restrictions, 4 smokers
C	10 participants	50-g chocolate bar	0, 15, 30, 45, 60, 90 and 120 min after consumption	Fasting caffeinated foods and drinks 12 h previous to the start of the experiment
D	6 participants	50-g chocolate bar or double espresso or neither (control)	0, 20, 40, 60, 90, 120 and 120 min after consumption	Fasting caffeinated foods and drinks 12 h previous to the start of the experiment
E	20 participants	Regular versus rare coffee drinkers	Sampled one time to check baseline levels	Fasting caffeinated foods and drinks 12 h previous to the start of the experiment

### Collection of sweat from the fingertip

Finger sweat samples were collected as previously published [32]. In short, filter papers were stamped out of fuzz-free paper to get a circular area of 1 cm^2^, filter papers were pre-wetted with 3 µl of LC–MS grade water and stored in 0.5-ml Eppendorf Tubes. For each sweat collection, donors cleaned their hands with warm tap water and then dried their hands with disposable paper towels. Donors kept their hands open in the air at room temperature for 1 min. Then, the sampling unit was placed between thumb and index finger with clean tweezers and held for 1 min. Filters were transferred back to labelled 0.5-ml Eppendorf tubes using clean tweezers and stored at 4 °C until sample preparation.

### Sample preparation

Each finger sweat sample collected on filter paper was extracted with 120 µl of extraction solution (an aqueous solution of 1 pg µl^−1^ caffeine-trimethyl-D9 with 0.2% formic acid). Metabolites were extracted via repeated pipetting of the extraction solvent 15 times. The filter paper was pelleted on the bottom of the Eppendorf Tube and the supernatant was transferred into HPLC glass vials equipped with a 200-µl V-Shape glass insert (Macherey–Nagel GmbH & Co.KG) and analysed by LC–MS/MS. To determine background metabolites or potential contaminants, blank filter papers were additionally extracted in the same manner. Standard solutions were prepared in methanol in a concentration of 1 mg ml^−1^. After that, they were diluted to concentrations of 100 pg µl^−1^ and 10 pg µl^−1^.

### *LC–MS/MS analysis*

Chromatographic separation was performed on a Vanquish UHPLC system (Thermo Fisher Scientific) and analysed in an untargeted fashion with a hybrid instrument consisting of a quadrupole mass filter and an orbitrap mass analyser (Q Exactive HF, Thermo Fisher Scientific). A reversed phase Kinetex XB-C18 column (100 Å, 2.6 µm, 100 × 2.1 mm, Phenomenex Inc.) was used to separate analytes present in finger sweat samples. Mobile phase A consisted of water with 0.2% formic acid and mobile phase B of methanol with 0.2% formic acid. The following gradient was applied: 1–5% B in 0.3 min and then 5–40% B from 0.3–4.5 min, followed by a column washing phase of 1.4 min at 80% B and a re-equilibration phase of 1.6 min at 1% B resulting in a total runtime of 7.5 min. Flow rate was set to 500 µl min^−1^ and the injection volume was 10 µl. Electrospray ionisation was performed in positive as well as negative ionisation mode. MS scan range was *m*/*z* 100–1000 and the resolution was set to 60,000 (at *m*/*z* 200) for MS1 and 15,000 (at *m*/*z* 200) for MS2. A top 4 method was applied and dynamic exclusion was set to 6 s. Collision energy was 30 eV. Instrument control was performed with Xcalibur software (Thermo Fisher Scientific).

### Data analysis

Raw files generated by the Q Exactive HF were searched in the Compound Discoverer Software 3.1 (Thermo Fisher Scientific) applying a user-defined workflow. All identified compounds with a match factor ≥ 80 were manually reviewed using Xcalibur 4.0 Qual browser (Thermo Fisher Scientific) and the obtained MS2 spectra were compared to reference spectra taken from *mzcloud* (Copyright © 2013–2020 HighChem LLC, Slovakia). A maximum retention time shift of 0.1 min was allowed and the mass tolerance was restricted to 10 ppm for MS1 and MS2. Identified compounds were also verified using purchased analytical standards applying the same LC–MS method. The Tracefinder Software 4.1 (Thermo Fisher Scientific) was used for peak integration and calculation of peak areas. Generated batch tables were exported and further process with Microsoft Excel, GraphPad Prism (for two-tailed, paired *t*-tests) and the Perseus [45] (for principal component analysis) software. ACD/Labs’ ChemSketch (Freeware) 2020.1.1 was used to draw structures.

## Results

### Both food and beverage consumption leave characteristic imprints on finger sweat composition and enable conclusions to be drawn about the general lifestyle of individuals

**Fig. 1:**
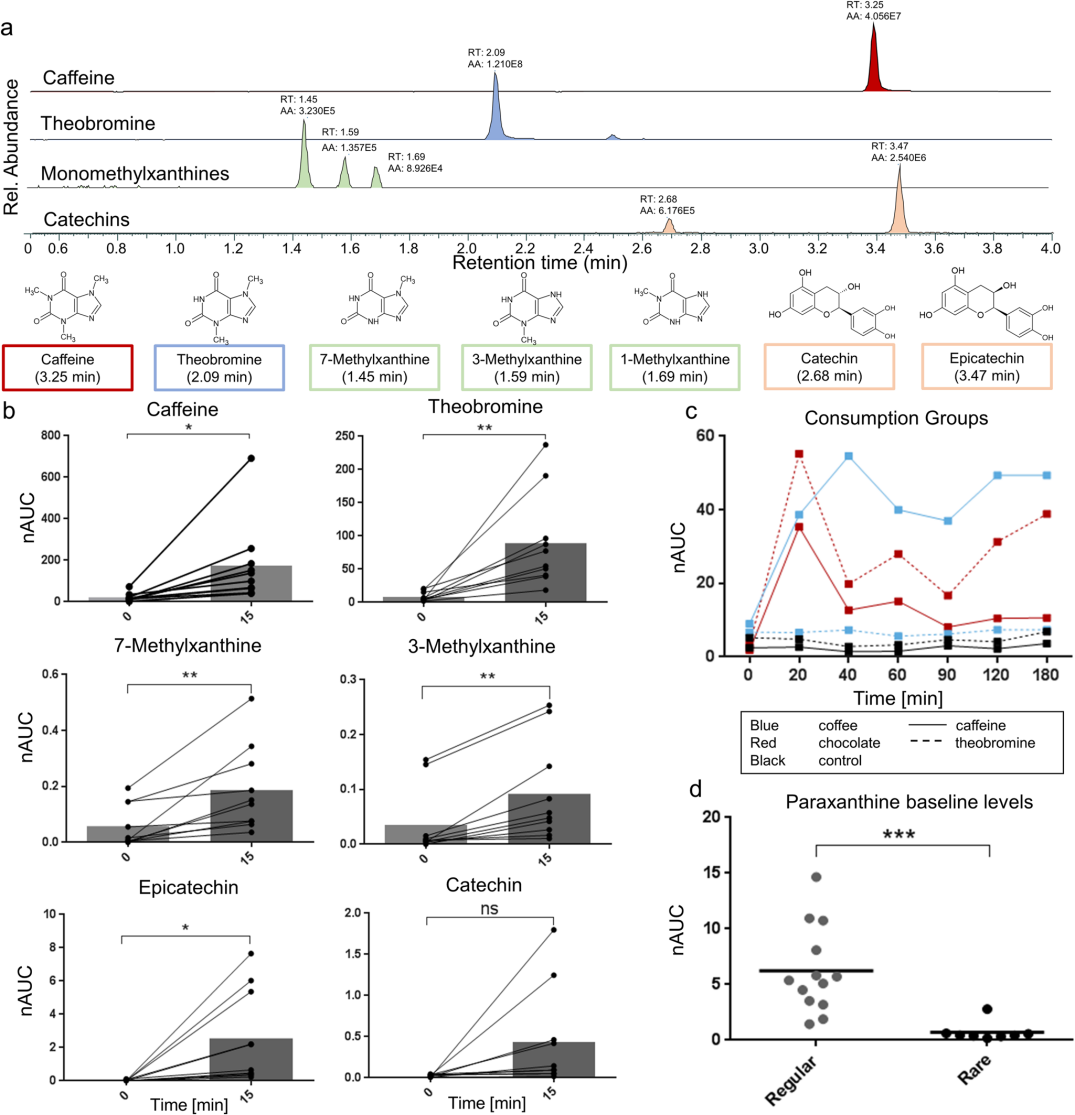
Imprints of food and beverages on finger sweat composition via high-resolution LC–MS/MS. **a** Characteristic metabolic profile in sweat after the consumption of chocolate exemplified by extracted ion chromatograms of caffeine (retention time (RT) = 3.25 min, *m/z* 195.0877, red peak), theobromine (RT = 2.09 min, *m/z* 181.0720, blue peak), 7-, 3- and 1-methylxanthine (RT = 1.45, 1.59 and 1.69 min, *m/z* 167.0564, green peaks) and catechin and epicatechin (RT = 2.68 and 3.47 min, *m/z* 291.0863 orange peaks) for a single donor. All of the shown molecules are known chocolate constituents or metabolites thereof, demonstrating that xenobiotics and their metabolites contained in foods and beverages can be identified in the sweat of volunteers after consumption. **b** Controlled study (study C) of 10 donors eating a 50-g chocolate bar. Normalised areas under the curve (nAUC) before (0 min) and 15 min after consumption are shown for caffeine, theobromine, 7-methylxanthine, 3-methylxanthine, epicatechin and catechin, demonstrating an increase in all individuals after 15 min. The increase was significant for all molecules except for catechin. *p-value ≤ 0.05, **p-value ≤ 0.01, ns not significant. **c** Time-course measurements of caffeine and theobromine shown for three donors consuming either a double espresso (blue), a 50-g chocolate bar (red) or neither (control, black) demonstrate an increase of those metabolites in a highly characteristic fashion, demonstrating that metabo-tip analysis can indeed identify consumption behaviour. nAUC, normalised area under curve. **d** Paraxanthine is the main metabolite of caffeine and accumulates in the sweat of regular coffee drinkers. Here, we show the comparison of paraxanthine baseline levels of regular (at least 1 cup of coffee per day) with rare coffee consumers, which allows the distinction based on the volunteers’ dietary preferences. ***p-value ≤ 0.001. nAUC, normalised area under the curve

Figure 1a shows extracted ion chromatograms identifying caffeine, 7-, 3- and 1-methylxanthine as well as catechin and epicatechin in a finger sweat sample of an individual 15 min after consumption of 50-g dark chocolate. None of these substances was detectable in the finger sweat of individuals before consumption; the significant increase of these compounds in finger sweat after chocolate consumption could be reproduced in a controlled study with ten different individuals (Fig. 1b). Furthermore, the levels of different caffeine metabolites after chocolate intake were compared to those after drinking a cup of coffee. Although both caffeine and theobromine are contained in chocolate as well as coffee, different levels of these two compounds found in finger sweat enabled the identification of the respective consumption groups (Fig. 1c). Paraxanthine is the catabolic metabolite of caffeine and apparently accumulates in regular coffee consumers [46, 47]. In our experiment, we could confirm this finding in individuals consuming coffee regularly through elevated baseline levels of paraxanthine in finger sweat (Fig. 1d). Thus, a metabolic property detected in finger sweat may be related to individual lifestyle preferences, such as coffee consumption. Obviously, nutrition is a major contributor to individual lifestyle habits. The monitoring of individual metabolic responses such as food intolerances upon the intake of specific foods and beverages as outlined below may gain great relevance in the further development of PPPM.

### Cigarette smoke, pharmaceuticals and exposure to environmental toxins leave characteristic imprints on finger sweat composition

**Fig. 2:**
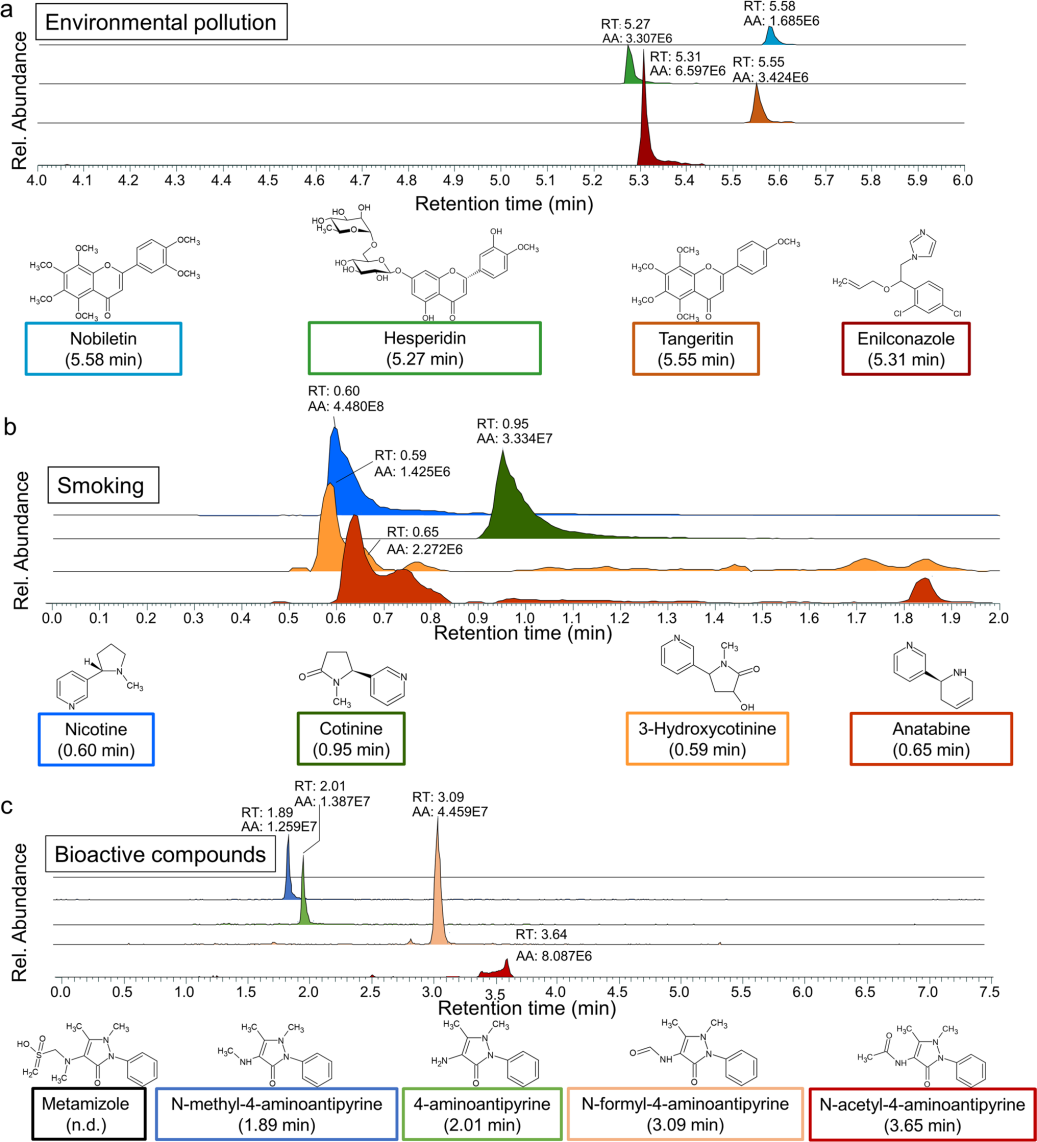
Metabo-tip reveals environmental pollution in nutrition, smoking habits and medication. **a** The intake of an orange (donor from study A) results in the detection of distinct metabolites in finger sweat. Extracted ion chromatograms are shown for nobiletin (RT = 5.58 min, *m/z* 403.1387, blue peak), hesperidin (RT = 5.27 min, *m/z* 611.1970, green peak), tangeritin (RT = 5.55 min, *m/z* 373.1282, orange peak) and enilconazole (RT = 5.31 min, *m/z* 297.0556, red peak), which is a fungicide predominantly used in the agriculture of citrus fruits. **b** The imprint of smoking on the finger sweat (donor from study B). Not only nicotine but also its metabolites were detected in sweat as well as anatabine, which is found in tobacco. The simultaneous detection of nicotine and its metabolites not only allows individual metabolic profiling but also rules out that nicotine is only present in finger sweat samples due to skin contamination by holding a cigarette. Extracted ion chromatograms of nicotine (RT = 0.60 min, *m/z* 163.1230, blue peak), cotinine (RT = 0.95 min, *m/z* 177.1022, green peak), 3-hydroxycotinine (RT = 0.59 min, *m/z* 193.0972, orange peak) and anatabine (RT = 0.65 min, *m/z* 161.1073, red peak) are depicted. **c** Evidence for the intake of a pain killer by the successful detection of its metabolites in finger sweat. Extracted ion chromatograms of metamizole (*m/z* 312.1013, empty trace) and its metabolites N-methyl-4-aminoantipyrine (RT = 1.89 min, *m/z* 218.1288, blue peak), 4-aminoantipyrine (RT = 2.01 min, *m/z* 204.1131, green peak), N-formyl-4-aminoantipyrine (RT = 3.09 min, *m/z* 232.1086, orange peak) and N-acetyl-4-aminoantipyrine (RT = 3.65 min, *m/z* 246.1237, red peak). Even though metamizole was not directly found in the volunteer’s sweat, metabo-tip analysis revealed the usage of the pain killer via the successful detection of the metamizole metabolites

**Fig. 3:**
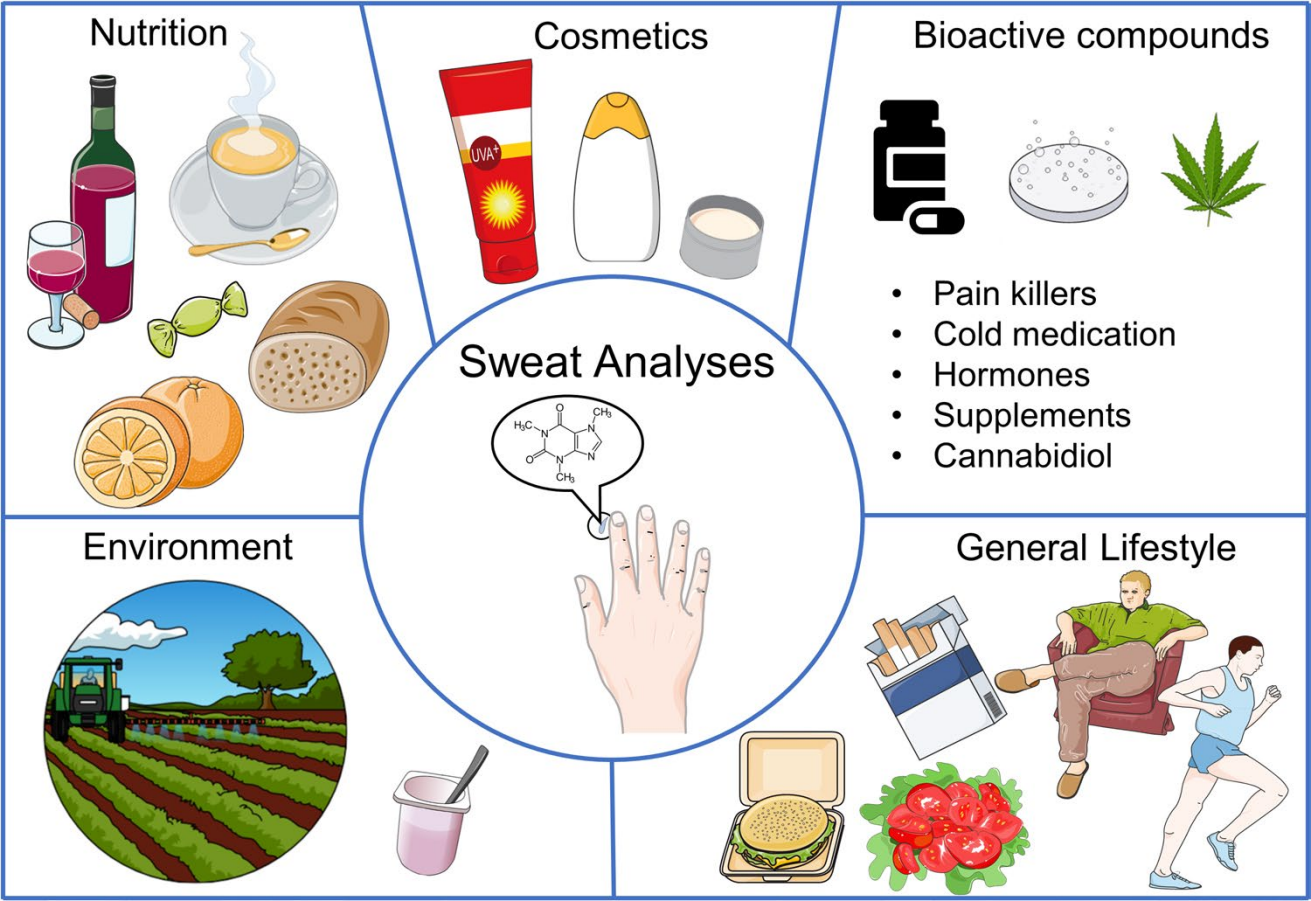
Monitoring lifestyle parameters via finger sweat analysis. Sweat used for metabo-tip analysis is collected by holding a filter paper between thumb and index finger without forcing sweat formation. Metabo-tip analysis reveals the highly dynamic nature of sweat composition and how it changes when specific foods and beverages are consumed (e.g. polyphenols found in chocolate and wine, xanthines like caffeine from coffee, flavonoids from citrus fruits and mycotoxins found in bread/wheats), when supplements for example vitamins or melatonin as sleep aid supplement are taken or when certain types of hygiene products have been used such as climbazole found in dandruff shampoos or oxybenzone from sunscreen lotions. Moreover, the general health status of a person can be assessed by revealing individual medication (e.g. for pain and fever, for colds, hormones, or cannabidiol) even allowing risk assessment for therapeutic strategies. Metabo-tip analysis may even reveal biomarker patterns associated with certain diseases or general lifestyle habits, like smoking. Exposure to environmental toxins derived from consumed products like pesticides (e.g. enilconazole, fenpropimorph or propamocarb) as well as toxic compounds in plastics found in the packaging of foods and beverages (e.g. bisphenol S and melamine) was also successfully detected in the sweat of test subjects. The Supplementary Table lists all compounds detected by metabo-tip analysis and shows the potential of metabo-tip analysis to support the development for novel PPPM strategies

Not only a specific diet but also personal habits such as smoking and regular use of pharmacological drugs will result in characteristic changes in finger sweat. Moreover, metabo-tip has the potential to detect even trace amounts of pesticides as demonstrated in Fig. 2. The consumption of an orange allowed for the identification of natural bioactive compounds such as flavonoids (nobiletin, hesperidin and tangeritin) together with pesticides such as enilconazole (Fig. 2a). In addition, metabo-tip was applied to detect smoking of tobacco (cigarettes) in finger sweat via characteristic compounds such as nicotine and anatabine (Fig. 2b). The simultaneous detection of precursor molecules and their metabolites such as nicotine, cotinine and 3-hydroxycotinine facilitates individual metabolic profiling (Fig. 2b). In the case that xenobiotics are not directly detectable, evidence can be found by the detection of specific metabolites. For example, the pain killer metamizole was not detectable in sweat, but after consumption of metamizole, a group of metamizole-derived metabolites could be detected in finger sweat, as demonstrated in Fig. 2c. The power of metabo-tip with respect to the detection of even trace amounts of substances with noxious effects, as summarised in Fig. 3, demonstrates its great potential as tool for PPPM supporting clinical practice. In this way, not only patient’s compliance regarding the intake of specific prescribed medications may be monitored but also a molecular risk assessment analysis upon the intake of toxins such as cigarette smoke can be performed.

### Sweat composition provides a wide spectrum of information regarding individual lifestyle

Metabo-tip not only allows the detection of specific compounds after consumption of a certain food or medication but also enables comprehensive, untargeted screening of exogenous bioactive compounds, for instance from cosmetics or environmental pollutants. In addition, it detects specific markers for general lifestyle habits (Fig. 3, Supplementary Table). Based on previously published studies [48, 49], it could be assumed that basic compounds are more likely to get transported into sweat ducts; however, our results do not seem to discriminate between analytes based on their pK_A_ values, therefore allowing for a comprehensive metabolic profile (Supplementary Table with listed pK_A_ values). The combined detection of exogenous compounds as well as endogenously produced metabolites results in a specific molecular signature in the finger sweat of each individual. Exposure to bioactive compounds as well as to various medications and drugs can be easily detected. Moreover, environmental pollutants such as fungicides, mycotoxins and compounds derived from plastic containers such as bisphenol S and melamine are detectable (Supplementary Table) and may be of great relevance concerning individual health. Thus, the wide array of information obtained from finger sweat analysis may reveal information about the exposition to toxins and general health status of an individual. This important property of metabo-tip may open up many remarkable opportunities for a more systematic investigation of exposure to toxins and individual responses thereupon, thus improving risk assessment and extending our opportunities to assess an individual’s health status.

### Metabo-tip analysis reveals diurnal fluctuations in metabolism and individual endogenous responses

**Fig. 4:**
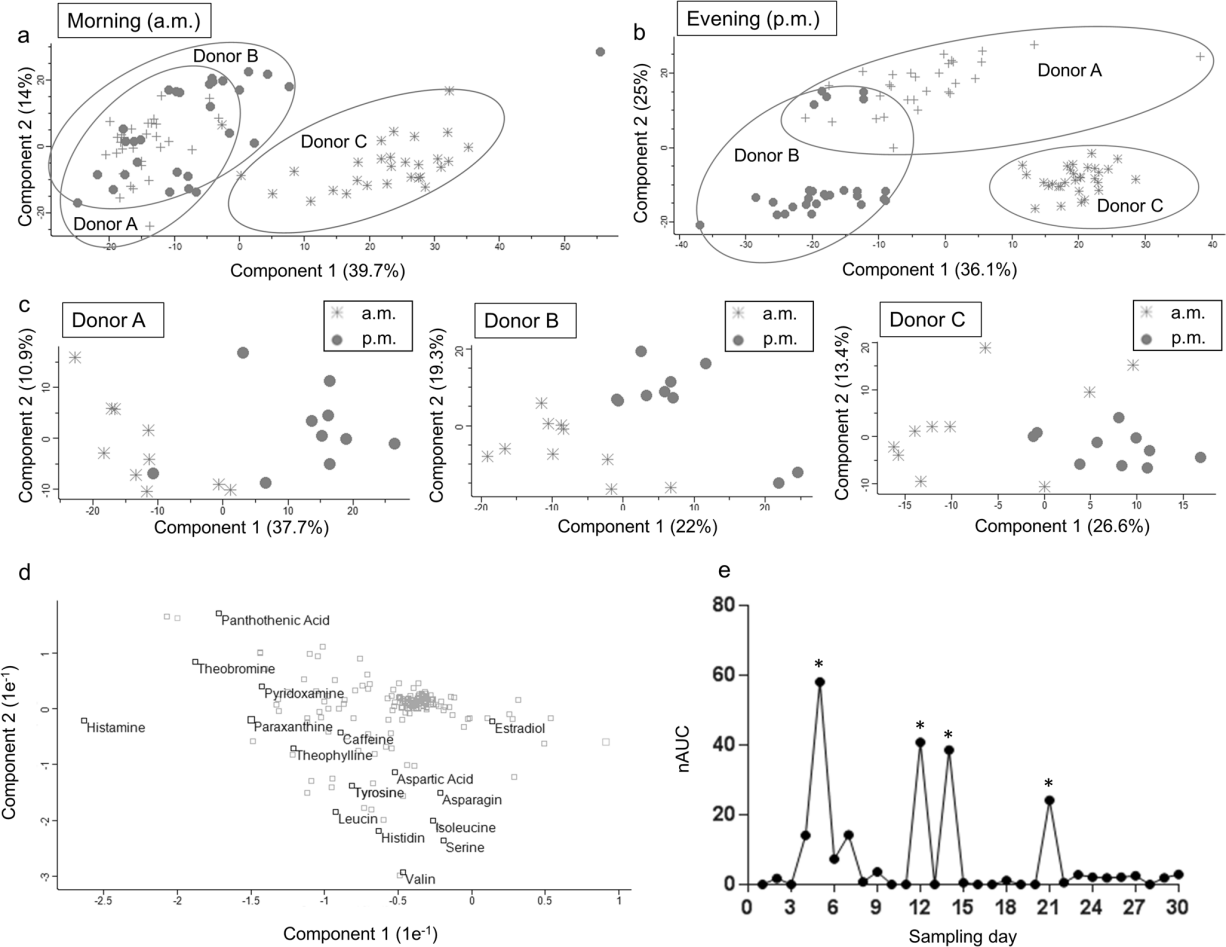
Diurnal metabolic fluctuations and individual endogenous responses. Principle component analyses (PCA) of finger sweat samples collected from three donors (study A) in the morning (**a**) and evening (**b**) over 30 days are depicted. Donor C can be completely separated from the other donors, while donors A and B share a rather similar lifestyle living in a joint household. **c** PCA of the finger sweat samples of three donors reveals diurnal metabolic fluctuations demonstrated by a consistent shift of component 1 to the right from morning to evening in each individual. **d** Loadings plot for the PCA depicted in **b**, showing the strong influence of histamine, coffee or chocolate consumption (caffeine, theobromine, theophylline and paraxanthine), and general diet (amino acids and vitamins like pantothenic acid) on components 1 and 2. Estradiol is a hormone and has only a minimal influence on components 1 and 2; however, this shows that further improvements of metabo-tip analysis may even allow to robustly distinguish male and female donors based on hormones as discriminators. **e** Time-course analysis of histamine over a time period of 30 days. Asterisk marks timepoints showing high histamine levels of volunteers accompanied by reported indispositions in relation to food or beverage intake

Principal component analysis (PCA) of finger sweat samples collected from three donors in the morning and evening over a time period of 30 days revealed fluctuating differences in finger sweat composition with the possibility to distinguish between these individuals based on their general lifestyle (Fig. 4a, b). Donor A and donor B could not be completely separated by PCA which might be contributed to a similar lifestyle as these donors share the same household. Intriguingly, a regular shift of metabolites in a diurnal rhythm was observed (Fig. 4c). Analysis of the PCA loadings plot suggested caffeine, theobromine, theophylline, paraxanthine and amino acids as most influential compounds (Fig. 4d).

This time series analysis was also used to assess the biogenic monoamine histamine, which serves both as a neurotransmitter and as a modulator in inflammatory responses. Figure 4e exemplifies the levels of histamine of an individual determined by metabo-tip analysis on consecutive days. Histamine peaks correlated with symptoms associated with food intolerance and allergy reported on the corresponding days. This observation indicated that specific individual responses to food consumption are detectable by metabo-tip analysis. Obviously, different individuals may respond rather differently to a given food, typically documented by gossips rather than molecular analyses. The capability of metabo-tip to detect individual molecular responses opens another highly relevant field of PPPM research.

## Discussion

Diagnosis and treatment of chronic disease are often difficult, because these diseases often have multiple causes—as evidenced in case of arteriosclerosis, type II diabetes, cancer or neurodegenerative diseases. Actually they may be caused or promoted by an imbalance of metabolic homoeostasis due to long-lasting stress. The investigation of metabolic imbalances in humans has been difficult though such investigation is crucial to predictive preventive personalised medicine (PPPM) improvement [50]. Due to its dynamic nature, metabolomic measurements call for repeated analyses in a narrow timeframe. Blood sampling is therefore hardly feasible as it leads to compliance issues. Non-invasive methods would be preferable but the analysis of urine, for instance, does not allow repeated time series analyses in a narrow timeframe because urine is collected in the bladder prior to sampling. Saliva may suffer from contaminants such as bacteria and food as well as from mucous glycoproteins which may interfere with metabolite extraction and which may impair sensitivity of measurement. Sweat is only available in limited amounts, and the risk of potential contamination from the skin also causes quantification challenges. To overcome some of these obstacles, we investigated sweat collected from clean fingertips via metabo-tip. This procedure is fast, non-invasive and easy to use, thereby supporting multiple measurements within a short timeframe, and ensuring good compliance by the test subjects [31]. A determination of individual enzymatic activities and a management strategy to cope with normalisation issues, related to the total amount of sweat collected per sample, has already been demonstrated [32]. Here, we evaluated whether this analytical method could detect specific substances that would reflect upon lifestyle habits which may be relevant to health to be determined.

### Extending current PPPM strategies by monitoring individual metabolic responses to the uptake of xenobiotics

We demonstrated that the uptake of as little as 0.2 mg of a chemical compound may be sufficient to be detected by metabo-tip analysis, and investigated if this approach was limited to basic compounds showing higher solubility in the slightly acidic sweat pH [49, 51]. Plotting the identified substances versus pK_A_ values did not show any relation, suggesting no discrimination of substances based on their pK_A_ (Supplementary Table). Thus, it seems that metabo-tip analysis may provide a representative overview of stable substances we are exposed to. Present data therefore suggest a broad applicability of metabo-tip. The detection of bioactive compounds such as flavonoids and even pesticides after the consumption of oranges (Fig. 2a) demonstrated the practicability of the assay.

Furthermore, the metabolism of xenobiotics could be observed using metabo-tip analysis as demonstrated in the case of xanthines, nicotine and metamizole (Figs. 1 and 2). Individual differences in the expression of P450 isoenzymes [52] may account for individual variations in drug effects and toxicity [53]. As demonstrated by us previously, proteomics and eicosanoid analysis revealed that the consumption of coffee may have significant pro- or anti-inflammatory effects in an individualised fashion [54]. In that study, age, sex, coffee consumption habits as well as the metabolic kinetics of caffeine in blood of each individual were ruled out as predictive parameters accounting for pro- or anti-inflammatory effects. Thus, the combination of different “-omics” approaches, especially the inclusion of metabolic profiling, may be of great relevance regarding PPPM and individual risk assessment [55]. As metabo-tip analysis allows for the profiling of xenobiotic exposure and the associated metabolic activities, it will greatly support more systematic studies to improve current PPPM strategies.

### Metabo-tip: a novel tool for the detection of endogenous metabolic responses

We have observed an apparent correlation between allergic symptoms and histamine detected by metabo-tip analysis (Fig. 4), which opens the opportunity to define biochemical signatures of some adverse effects. Metabo-tip analyses will allow us to investigate apparent detrimental effects with scientific rigour, enabling reasonable conclusions to be made regarding risk assessment. Furthermore, the presented analytical workflow offers the opportunity to monitor individual responses to plant-derived bioactive compounds, especially flavonoids, which are intensely discussed in the context of PPPM because of their beneficial impact on carcinogenesis [56, 57]. Specifically, the detection of histamine points to the enormous power of metabo-tip for clinical studies related to food intolerance. So far, only volunteers reporting no acute medical disorders were included in our studies. Thus, a systematic and large-scale evaluation of the predictive power of metabo-tip analysis in patients suffering from various medical disorders needs to be performed in order to evaluate the power of metabo-tip for clinical routine applications.

The opportunities offered by metabo-tip analysis are raising many questions. Only large-scale studies will allow us to decide, e.g., whether the apparent diurnal rhythm observed by metabo-tip in this study (Fig. 4) was related to endogenous factors (e.g. through circadian regulation), or rather exogenous factors (such as regular nutrient patterns or repeated exposure to xenobiotics). Furthermore, technical improvements regarding robustness, sensitivity and throughput may also support the development of stress monitoring devices as well as therapy monitoring strategies, bringing about crucial progress in the field of PPPM.

### Sweat may serve as specific source for reactive oxygen species-associated metabolites

We have recently reported that erythrocytes interfere with reactive oxygen species (ROS) formation so efficiently that ROS-dependent T-cell activation is blocked by the mere presence of erythrocytes [58]. This observation clearly implies that the formation of ROS-dependent metabolites in blood may be inhibited as well. Inflammatory activation of leukocytes typically takes place in the interstitium, and actually sweat reflects the constitution of interstitial fluid [59, 60] which is free from erythrocytes. Therefore, the analysis of sweat may provide the advantage of including ROS-associated metabolites in the analysis which may otherwise be missing in blood. On the other hand, labile and reactive metabolites may react already at the skin surface due to the presence of oxygen and get lost for meaningful evaluation. These aspects highlight the huge potential of metabo-tip only to be realised by more basic research.

### Ways to translate metabo-tip into clinical practice and its potential impact on supporting PPPM concepts

Up until now, metabo-tip was exclusively used in the framework of basic research projects to identify and uncover its power and applicability in various research areas. In this context, a comprehensive metabolomic screening approach was conducted which requires specially trained personnel in the field of LC–MS/MS and data analysis. The translation of metabo-tip analysis as tool for PPPM in the clinical routine will require some practical adaptions. Based on the screening approach, a panel of molecules serving as biomarkers should be selected for targeted analysis, facilitating data management and evaluation. Regarding the general cost–benefit of metabo-tip analysis in clinical practice, it will prove most cost-effective in the long run since the principal costs will fall to the purchase and installation of a suitable LC–MS/MS system.

Thus, a large number of PPPM-related applications, potentially offered by metabo-tip analysis, warrant larger scale studies. The non-invasive and painless sampling procedure in combination with high-throughput analysis enables the systematic monitoring of individuals in short intervals. Hence, the opportunity of frequent sampling makes it even possible to carry out specific metabolic in vivo studies in humans. Up until now, the most comprehensive metabolic studies were conducted using in vitro cell culture model systems. As demonstrated previously, those experiments often suffer from unexpected influencing factors such as the composition of fetal calf serum [61]. The advantage of metabo-tip analysis is the facilitated use of humans as model system for comprehensive metabolic studies in vivo. This possibility enables the monitoring of a plethora of metabolic intervention studies in individuals in the context of PPPM such as the monitoring of food intolerances, the examination of individual lifestyle changes in association with the general health status and risk assessment and the monitoring of the intake of prescribed medications in clinical practice.

## Strengths and limitations

Metabo-tip analysis is a fast, non-invasive, painless and easy to use strategy, thereby supporting metabolic measurements in a short timeframe, and ensuring optimal compliance by the test subjects. These strengths enable the individualised monitoring of metabolites and the assessment of metabolic kinetics of individuals as response to various stimuli. Nevertheless, some limitations of metabo-tip analysis have to be considered. Up until now, metabo-tip was applied for basic research projects only. The applicability of metabo-tip in clinical routine has still to be evaluated systematically by setting up targeted LC–MS/MS methods applied to larger study cohorts. The small amount of sweat used for metabo-tip analysis requires highly sensitive analytical approaches for metabolomic analyses with the drawback that even small contaminations on the fingertip of test subjects can already impede and distort the analysis. Furthermore, the absolute quantification of metabolites represents a true challenge. However, using the kinetic profile of biochemically related pairs of molecules such as caffeine and its metabolites, i.e. xanthines, has already been proven to support normalisation and determine individual sweat flux rates.

## Conclusions and outlook

We conclude that metabo-tip offers a great opportunity to support the further development of PPPM strategies due to its non-invasive character and the possibility of painless sampling in a narrow timeframe while providing rich molecular information. In this way, personalised molecular profiles can be generated allowing us to observe individual metabolic reactions to exogenous and/or endogenous stimuli or challenges. Ongoing research will establish the relation of individual molecular patterns obtained by metabo-tip with disease states or risk for diseases. We thus suggest that metabo-tip may have a large number of clinical applications ranging from diagnostic applications such as the early detection of food intolerances to the monitoring and validation of therapeutic strategies.

## Supplementary Information

Below is the link to the electronic supplementary material.

Supplementary file1 (XLSX 23 KB)

## Data Availability

Raw data is secured and available on request.

## Abbreviations

CV: Coefficient of variation
LC–MS: Liquid chromatography mass spectrometry
*m/z*: Mass over charge
nAUC: Normalised area under the curve
ns: Not significant
PCA: Principle component analysis
pK_A_: Acid dissociation constant
PPPM: Predictive preventive personalised medicine
ROS: Reactive oxygen species
RT: Retention time
